# Highly Sensitive Polydiacetylene Ensembles for Biosensing and Bioimaging

**DOI:** 10.3389/fchem.2020.565782

**Published:** 2020-11-13

**Authors:** Qiong Huang, Wei Wu, Kelong Ai, Jianhua Liu

**Affiliations:** ^1^Department of Pharmacy, Xiangya Hospital, Central South University, Changsha, China; ^2^Department of Geriatric Surgery, Xiangya Hospital, Central South University, Changsha, China; ^3^National Clinical Research Center for Geriatric Disorders, Xiangya Hospital, Central South University, Changsha, China; ^4^Xiangya School of Pharmaceutical Sciences, Central South University, Changsha, China; ^5^Hunan Provincial Key Laboratory of Cardiovascular Research, Xiangya School of Pharmaceutical Sciences, Central South University, Changsha, China; ^6^Department of Radiology, The Second Hospital of Jilin University, Changchun, China

**Keywords:** polydiacetylene, self-assembly, biological detection, colorimetric detection, bioimaging, fluorescence, Raman

## Abstract

Polydiacetylenes are prepared from amphiphilic diacetylenes first through self-assembly and then polymerization. Different from common supramolecular assemblies, polydiacetylenes have stable structure and very special optical properties such as absorption, fluorescence, and Raman. The hydrophilic head of PDAs is easy to be chemically modified with functional groups for detection and imaging applications. PDAs will undergo a specific color change from blue to red, fluorescence enhancement and Raman spectrum changes in the presence of receptor ligands. These properties allow PDA-based sensors to have high sensitivity and specificity during analysis. Therefore, the PDAs have been widely used for detection of viruses, bacteria, proteins, antibiotics, hormones, sialic acid, metal ions and as probes for bioimaging in recent years. In this review, the preparation, polymerization, and detection mechanisms of PDAs are discussed, and some representative research advances in the field of bio-detection and bioimaging are highlighted.

## Introduction

Biomolecule self-assembling is highly implicated in various biologic events through a variety of non-covalent interactions, such as hydrophobic interaction, hydrogen bonds, electrostatic interaction, and metal coordination (Bera et al., 2020; Yu et al., 2020). The self-assembly structures are the basis of forming different complex biological structures (Cao et al., 2020). For example, the cell membrane is mainly an elastic semi-permeable membrane composed of amphiphilic phospholipids (small organic molecules with a hydrophilic phosphate heard and a hydrophobic long alkyl chain tail) (Momcilovic et al., 2020). Inspired by the self-assembly events in living organisms, scientists have prepared many functional supramolecular systems through the self-assembly of organic small molecules in recent years (Ballandras-Colas et al., 2017; Chen et al., 2018; Vantomme and Meijer, 2019). Among them, the amphiphilic diacetylene-based self-assembly systems have attracted great attention in the field of basic and applied research (Lu et al., 2001; Peng et al., 2009; Hu et al., 2014; Chae et al., 2016; Ishijima et al., 2017a; Jordan et al., 2017; Nsubuga et al., 2018; Ortiz-Cervantes et al., 2018). Amphiphilic diacetylenes not only have the self-assembly properties similar to phospholipids, but more importantly, it can form a conjugated structure through polymerization (Wang et al., 2017). The covalently connected conjugated structures can give PDAs far beyond the stability of common supramolecular self-assembly (Park et al., 2016a; Tian et al., 2020). The stable structure is very important for biological detection and bioimaging, especially for complex environments in organisms.

**Figure 1:**
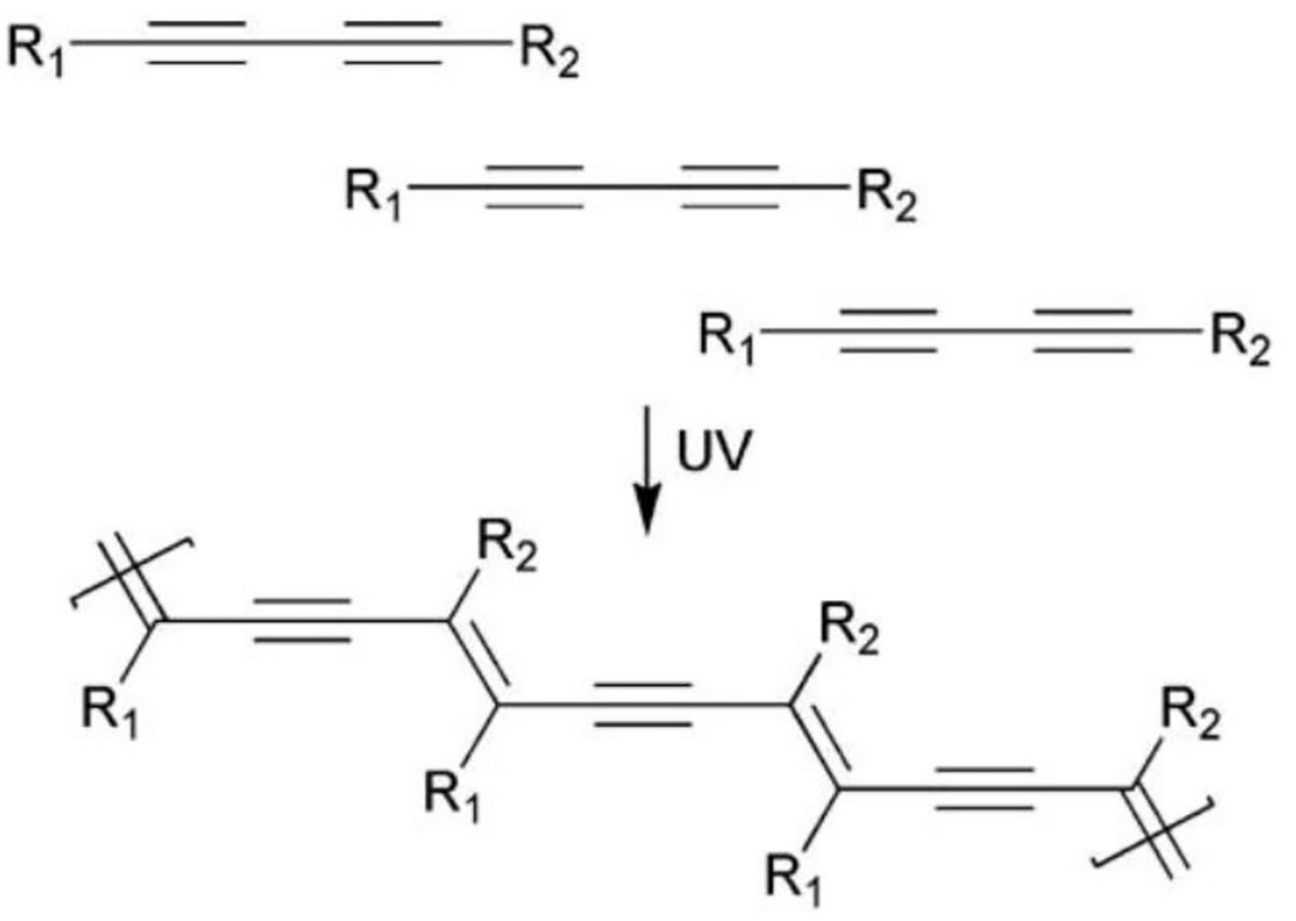
Topochemical photopolymerization of diacetylene. Reprinted with permission from Reppy and Pindzola (2007). Copyright 2007, Royal Society of Chemistry.

A series of amphiphilic diacetylene molecules with different polar heads and tail groups can be prepared by the Cadiot-Chodkiewicz reaction using acetylene or halocetylene derivatives (Reppy and Pindzola, 2007). The topochemical polymerization takes place among the arranged diacetylene amphiphiles under excitation of the ultraviolet light or gamma ray, followed by the formation of polymer conjugated skeletons with alternating C=C and C=C bonds (Figure 1) (Reppy and Pindzola, 2007; Li et al., 2018). The special conjugated main chain of PDA gives it unique C=C Raman signal, strong absorption at 640 nm, and fluorescence properties. PDAs are easy to be functionalized and can specifically bind to specific biomolecules. The absorption spectra of the functionalized PDAs would migrate from 640 to 540 nm after binding. This process will cause the color change of PDA from blue to red, coupled with the appearance of red fluorescence. These characteristics make PDA very attractive for applications in biological detection and bioimaging.

In 1993, Charych et al. reported the first case of modified PDAs for detection of virus (Charych et al., 1993). During the following 20 years, many PDA-based bioprobes have been developed to detect biomarkers, biomolecules, metal ions, drugs, proteins, nucleic acids, lipids, virus, bacterial, and use to bioimaging (Chae et al., 2016; Park et al., 2016b; Qu et al., 2016; Yamamoto et al., 2016; Jiang et al., 2017; Cui et al., 2018a; Kang et al., 2018; Nsubuga et al., 2018; Stauber et al., 2018; Takeuchi et al., 2018; Heo et al., 2019; Jung and Park, 2019; Kim and Lee, 2019; Oh et al., 2019; Romero-Ben et al., 2019; Wang et al., 2019a; Zhang et al., 2019a; Adhikary et al., 2020; Fang et al., 2020; Hao and Zhu, 2020; Li et al., 2020; Tian et al., 2020). Our research group has also carried out some related work in this field. For example, we used imidazole-functionalized PDA nanosheets to detect lysophosphatidic acid (an early-stage ovarian cancer biomarker) in serum (Wang et al., 2017). In recent years, the preparation methods for PDA have been greatly advanced. PDA structures have been enriched from traditional films, micelles and vesicles to 3D-PDA structures, porous structures, hydrogels, macrocyclic structures, and single-layer nanosheets (Bang et al., 2016; Chae et al., 2016; Krishnan et al., 2016; Okaniwa et al., 2016; Jordan et al., 2017; Li et al., 2017a; Romera et al., 2017; Jeong et al., 2018; Takeuchi et al., 2018; Terada et al., 2018; Heo et al., 2019; Hu et al., 2019; Seo et al., 2019; Shin et al., 2020). Great progresses have also been made in the preparation of reversible and chiral PDA (Chen et al., 2016; He et al., 2020). PDA bioprobes based on these novel structures can further increase the sensitivity and reproducibility of detection, and their detection applications have thus been expanded to a wide range of biomolecules. For example, PDA colorimetric and fluorescent probes have been created to detect transmembrane action in the blood brain barrier (Adhikary et al., 2020), drug carrier efficiency (Wang et al., 2019a), chiral recognition (He et al., 2018) etc. In addition, PDA probes have also been extended to the fields of Raman bioimaging and Raman detection (Cui et al., 2018a; Tian et al., 2020). In this paper, we first introduce conventional and advanced preparation technologies of PDA. On this basis, the applications, and the latest progress of PDA in biological detection and bioimaging is highlighted. Finally, the challenges and opportunities for practical applications of PDA in biomedical field are discussed.

### Preparation of PDA

Diacetylene amphiphile is generally composed of a hydrophilic head and a hydrophobic tail. The tail can be divided into three parts: a long chain fat chain in the end, a diacetylene group, and a spacer between diacetylene. Conventional structures of PDA include Langmuir films, Langmuir-Blodgett films, self-assembled monolayers, etc. (Jiang and Jelinek, 2014; Samyn et al., 2014; Ariza-Carmona et al., 2015a,b; Araghi and Paige, 2017; Garcia-Espejo et al., 2017). PDA Langmuir film is a regular monolayer membrane formed by self-assembly of diacetylene amphiphile molecules at the oil-water interface, while PDA Langmuir-Blodgett film is a double-layer film formed by transferring PDA Langmuir film from oil-water interface to solid interface. However, PDA self-assembled monolayers are generally ordered ultrathin films formed by chemical adsorption at the solid-liquid interface. For example, the diacetylene amphiphiles modified by sulfhydryl groups are adsorbed on the gold surface to form a regular monolayer, and PDA self-assembled monolayers would be obtained after polymerization.

Common PDA structures for biosensors exhibit spherical, linear, and two-dimensional lamellar structures. For example, Baek et al. prepared PDA spherical nano-vesicles with an adjustable particle size from 36 to 84 nm by the microfluidic chip method (Baek et al., 2016). van der Asdonk et al. prepared PDA nanowires from the micron to centimeter scale using liquid crystal template (van der Asdonk et al., 2016). Recently Hu et al. prepared PDA nanosheets with an one-layer thickness (Hu et al., 2019). They first adopted a novel diacetylene amphiphile molecule: maleic acid as the head, the hydrophobic diacetylene as the tail, and 1,1,3,3-tetramethylyuanidine as the linker. After self-assembly of diacetylene amphiphiles, monolayer PDA was prepared by polymerization. Ishijima et al. also developed a general method to prepare two-dimensional PDA with the thickness of nanometer (Ishijima et al., 2017b). They used a specific stripping method to prepare PDA with 5 nm thickness in aqueous and non-polar organic solutions. However, the sensitivity and repeatability of bioprobes based on these PDA structures remains unsatisfactory. For instance, PDA probes usually have a good reversibility in response to environmental factors, but there are still a lot of room for improvement in the reversibility of biomolecules. In recent years, many scientists have made great efforts to solve these problems by introducing three-dimensional (3D)-structure or porous PDA, chiral PDA, and reversible PDA.

### 3D or Porous PDA

Compared to one-dimensional or two-dimensional structures, 3D or porous PDAs can significantly improve the detection sensitivity, because 3D or porous structures have more active sites to interact with biomolecules. For this reason, ever-growing biosensors based on 3D or porous PDAs have been developed in recent years.

**Figure 2:**
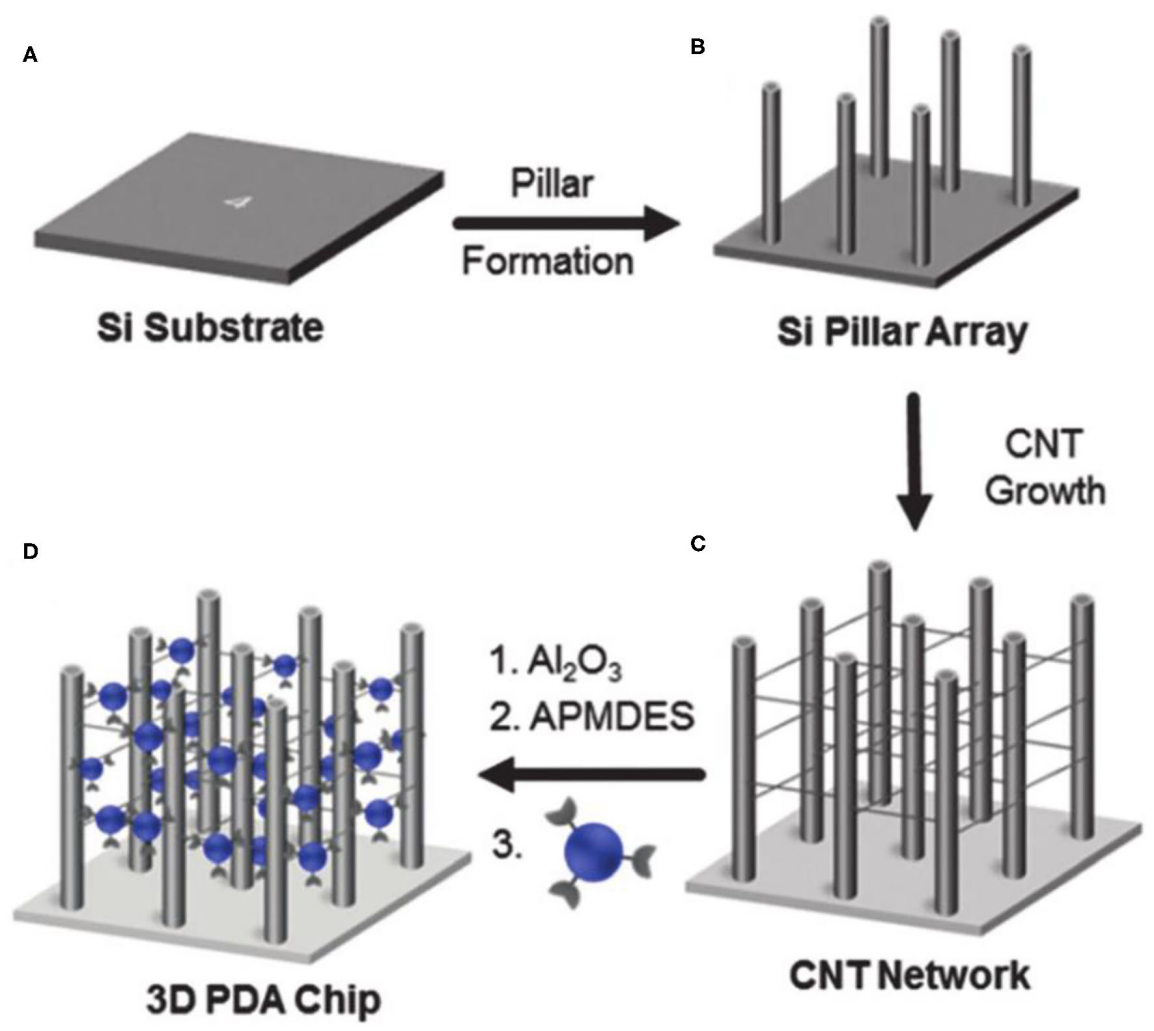
Schematic illustration of the fabrication of the 3D networked PDA sensor. Reprinted with permission from Lee et al. (2019a). **(A)** A p-type silicon subsrate. **(B)** Micropillar structures were prepared on a p-type silicon (100) substrate by a silicon deep etching process. **(C)** Networked carbon nanotubes (CNTs) were fabricated. **(D)** The 3D CNT networked pillared structures were coated with Al2O3 using an atomic layer deposition method. Copyright 2016, Royal Society of Chemistry.

Soobum Lee et al. constructed a PDA sensor system with a 3D network structure on the p-type silicon substrate (Lee et al., 2016a). As shown in Figure 2, they first prepared the micro-column array on the p-type silicon (100) substrate by silicon deep etching process, and then constructed the network with carbon nanotubes on the micro column array. A layer of Al_2_O_3_ was coated on the 3D substrate by atomic layer deposition, followed by modification with 3-amino-propylmethyldiethoxysilane. After further conjugation with 10,12-pentacosadienoic acid, 3D PDAs were obtained upon polymerization. The sensitivity of the engineered 3D network PDA sensor was improved by more than three orders of magnitude compared with traditional 2D-PDA sensors. However, the preparation method requires multi-steps and thus is relatively complex. To address this limitation, Hao Jiang and others developed a simpler method for preparing PDA with hierarchical nanostructures driven by divalent metal ions (Jiang and Jelinek, 2016). The diacetylene amphiphiles with melanin as the head were mixed with 10,12-tricosadyinoic acid in the aqueous solution. By introducing divalent zinc ions, the mixture of diacetylene amphiphiles began to form vesicles, followed by self-assembly into nanotubes and spontaneously cross-linking to form hierarchical nanostructures. The obtained PDA biosensors demonstrated high sensitivity to bacteria.

**Figure 3:**
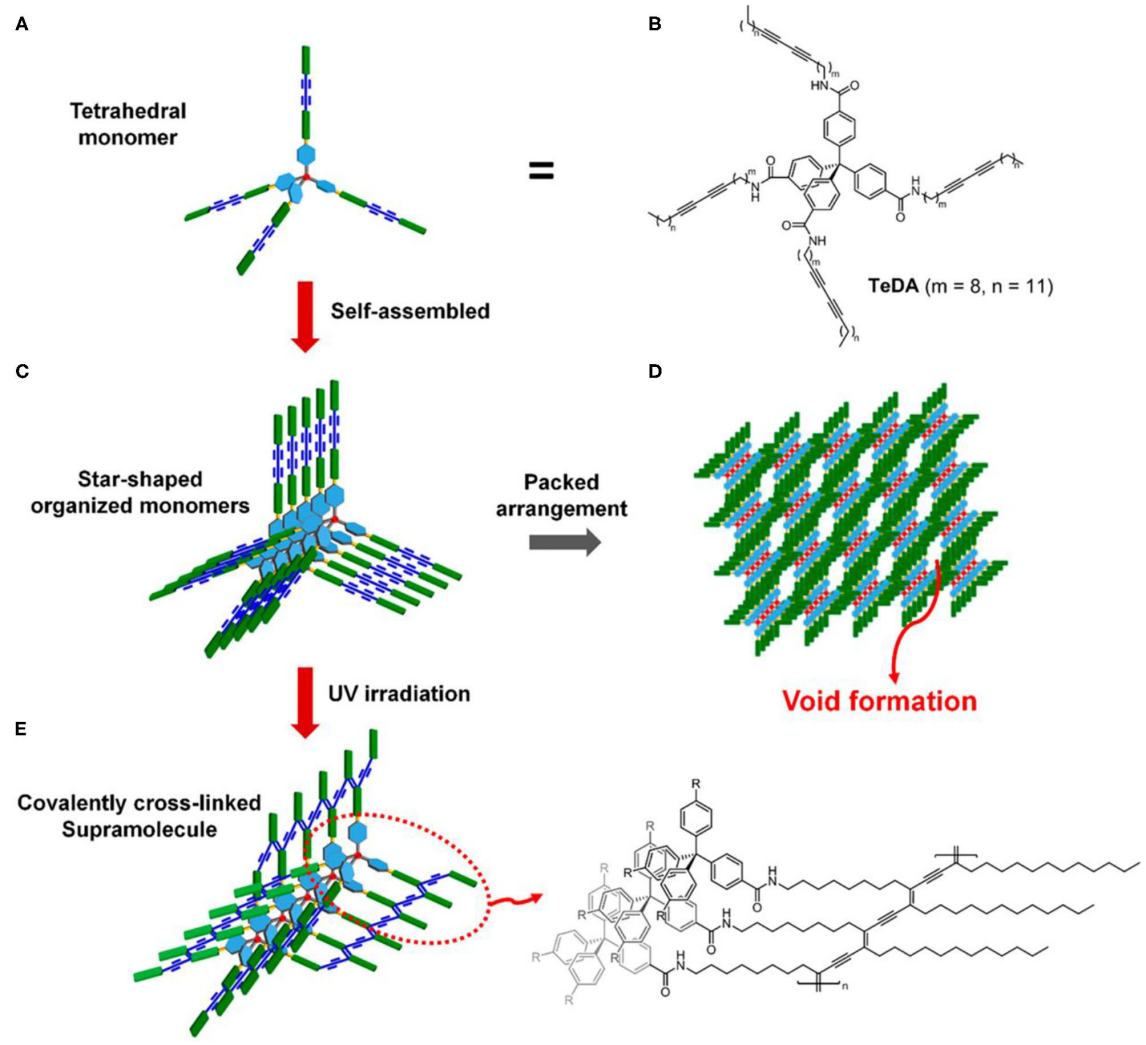
Illustration of porous polymer formation from self-assembled tetrahedral diacetylene. **(A)** Schematic representation of tetrahedral diacetylene. **(B)** Structure of the tetrahedral diacetylene. **(C)** Self-assembly of monomeric tetrahedral diacetylene. **(D)** Formation of the porous structure derived from the arrangement of monomer units. **(E)** Topochemically polymerized tetrahedral diacetylene and its structure. Reprinted with permission from Jeong et al. (2018). Copyright 2018, American Chemical Society.

Generation of molecular level porous structure in PDA materials can improve the detection sensitivity of PDA-based probes to a greater extent. However, it is difficult to produce porous PDA by self-assembly of common linear diacetylene amphiphile molecules. In order to obtain porous PDAs, diacetylene amphiphiles must possess several key structural characteristics, such as rigidity, space, and molecule-level twisted structure, to prevent the intermolecular and intramolecular dense packing. Recently, Jeong et al. constructed PDA with an endogenous porous structure by using the tetraphenylmethane-attached diacetylene derivative (Figure 3) (Jeong et al., 2018). The tetraphenylmethane diacetylene had rigid and twisted structures at the molecular level, so they could prevent intermolecular and intramolecular dense packing of molecules. After topochemical polymerization, porous PDA structures composed of tetraphenylmethane polydiacetylene was prepared. The tetrahedral PDA had mesoporous structure with an average pore size of 20.417 nm and a specific surface area of 5.5927 m^2^/g.

### Chiral PDA

Chirality is ubiquitous in nature. Chiral materials, such as chiral conjugated polymers, have been widely used in quantum computing, biological detection based on chiral recognition, chiral optical materials, and other fields (Gliga et al., 2017; Morrow et al., 2017; Kim and Kotov, 2019; Zhang et al., 2019b). In the past few decades, many strategies have been developed for synthesis of chiral conjugated polymers, mainly using chiral monomers, dopants, catalysts, circularly polarized light, vortex motion, agitation, and other physical forces as symmetry breaker (Xu et al., 2014; Gliga et al., 2017; Orlova et al., 2018; Wang et al., 2019b; Gagnon et al., 2020).

Given the unique features of chiral materials, chiral PDAs are also prepared by self-assembly of diacetylene amphiphile with chiral head groups or the introduction of chiral molecules as chiral guiding reagents. For example, Zhong et al. prepared chiral PDA Langmuir-Blodgett films with gemini type amphiphilic diacetylene derivatives (Zhong et al., 2010). In 2016, Chen et al. found that chiral L-histidine ester derivative LHC18 amphiphiles could not only help the formation of diacetylene amphiphiles gels, but also endow PDA polymers with chiral properties (Chen et al., 2016).

**Figure 4:**
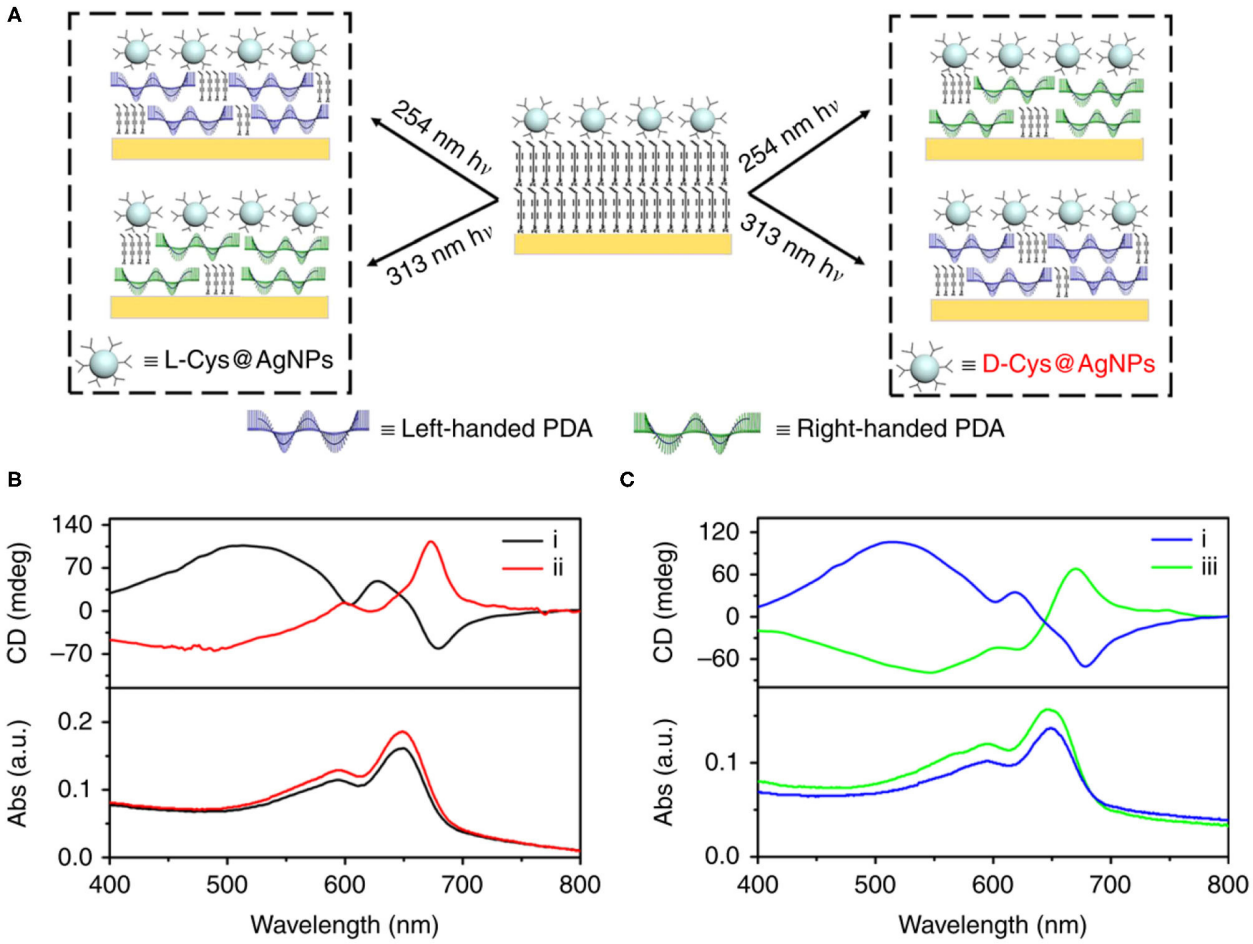
**(A)** Handedness of PDA depending on both chiral configuration of ligands on plasmonic nanoparticles (L- vs. D-) and the wavelength of UV irradiation (254 vs. 313 nm). Handedness of PDA obtained with all four possible combinations. Left-handed PDA was obtained using 254 nm irradiation assisted with L-Cys@NPs or 313 nm irradiation assisted with D-Cys@NPs. Right-handed PDA was obtained using 254 nm or 313 nm irradiation assisted with L-Cys@NPs. **(B)** CD spectra showing that chirality of PDA was reversed using (i) L- and (ii) D-Cys@AgNPs upon irradiated with 254 nm unpolarized light. **(C)** CD spectra showing that chirality of PDA was reversed using (iii) 313 nm irradiation instead of (i) 254 nm unpolarized light in the presence of L-Cys@AgNPs. Reprinted with permission from He et al. (2020). Copyright 2020, Nature.

On the other hand, the circularly polarized light is believed to be a possible origin of chirality of natural molecules (Tang and Cohen, 2011; Kim J. et al., 2015). Therefore, chiral PDAs can also be prepared by circularly polarized light. For example, Zou et al. employed the circularly polarized light to prepare PDA Langmuir-Blodgett films in 2009 (Zou et al., 2009). However, only a small amount of enantiomeric excess (<4%) were obtained with this method due to their small anisotropy factor (Meinert et al., 2014), leading to the low efficiency in the preparation of chiral PDA. To solve this problem, He et al. synthesized chiral PDA with a high efficiency by using the so-called super circularly polarized light instead of circularly polarized light (He et al., 2018). Super circularly polarized light was formed by the interference of two circularly polarized light propagating in the same frequency and opposite direction. They found that the application of super circularly polarized light could greatly improve the enantioselective polymerization of the achiral diacetylene monomer, with the maximum enhancement of about 6 times compared to the ordinary circularly polarized light. Moreover, the enantioselective synthesis of helix PDA chain could be strictly controlled. However, the super circularly polarized light only formed in a specific region, significantly limiting in the preparation of chiral PDA. Recently, it has been found that the chiral response could also be enhanced by noble metal nanoparticles with surface plasma enhanced effect. He et al. prepared the high chiral asymmetry PDA with cysteine-modified silver nanoparticles as the chiral inducer under excitation of non-polar UV light (Figure 4) (He et al., 2020). In addition, they found that the helicity of chirality could be controlled by changing the wavelength of UV light. This method provided a more economic and powerful method for preparation of programmable two-dimensional chiral PDA, such as smooth gradient of chirality and micro pattern with customizable circularly polarized luminescence.

### PDA With Reversible Response

At present, most PDA based bio-probes are not reversible. When the responsive biomolecules are removed, the color of PDA cannot be restored. The basic reason is that the binding force between the side chains of PDA is not strong enough, resulting in its inability to restore the conformation of the original conjugated PDA form. For this reason, one of promising strategies to prepare PDA with reversible responses is to improve the binding force between the side chains of PDA.

**Figure 5:**
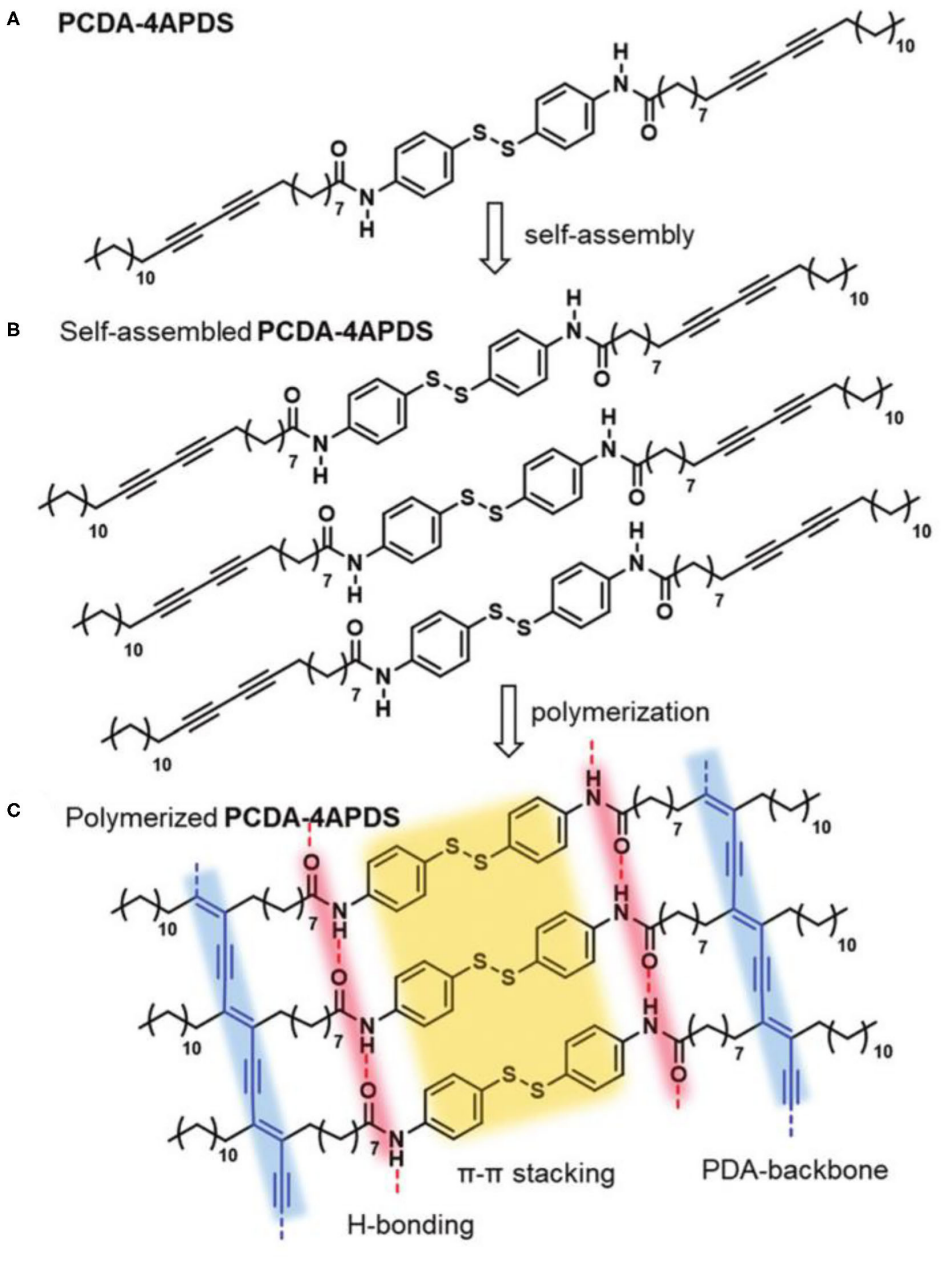
**(A)** Chemical structure of the diacetylene monomer, PCDA-4APDS. **(B)** Schematic illustration of molecular self-assembly of PCDA-4APDS. **(C)** Schematic representation of polymerized PCDA-4APDS showing intermolecular p–p stacking and H-bonding interactions, and the poly(ene–yne) backbone of the PDA. Reprinted with permission from Lee et al. (2019a). Copyright 2019, Royal Society of Chemistry.

Lu et al. used Gly–Ala–Gly–Ala–Gly–Ala–Gly–Tyr polypeptide chain at the polypeptide chain at the head of diacetylene amphiphiles to enhance the binding force between the side chains of PDA (Lu et al., 2016). Owing to the multiple hydrogen bonds between the polypeptide chain groups, the binding force between the side chains of PDA was greatly enhanced, thus enabling the PDA fiber to reversibly response to the targets. Lee et al. also reported the preparation of reversibly responsive PDA using diacetylene amphiphiles with highly rigid diphenyl disulfide head (PCDA-4APDS) (Figure 5) (Lee et al., 2019a). The Hydrogen bond and π-π stacking interactions coexist in the side chain of PDA, which could greatly enhance the side chain binding force of the PDA to endow PDA with a reversible response. The large ring structure was also adopted to prepare PDA with reversible responses to analyses. Recently, Shin et al. prepared column structure PDA by macrocyclic diacetylenes with the pyridine group (Shin et al., 2020). The covalent bond was used to connect the stacking elements along the column axis, leading to significant enhancement in the rigidity of the PDA and subsequent reversibly responsive capability.

## PDA for Colorimetric Detection

Generally, PDA bioprobes are prepared by functionalizing the PDA side chains with groups that can specifically bind to biological molecules. When biomolecules interact with PDA probes, the color of PDA probes will change from blue to red. In this way, biomolecules can be detected by colorimetry. It is therefore important to study the color change of PDA probes for the design of sensitive PDA probes. Alkyne carbons in diacetylene amphiphile monomers are sp hybrid with a bond angle of 180 degrees. Polymerization of diacetylene amphiphiles leads to the transformation of alkyne carbons from sp hybrid to sp^2^. Under normal condition, the bond angle of sp^2^ hybrid is 120 degrees. However, the force between the closely packed side chains makes PDA unable to rotate and change the angle of the PDA main chain during polymerization, resulting in stress in PDA. The biological interaction on the side chain of PDA sensors can cause the change of packed side chain, thus releasing the stress and enabling the main chain of PDA to rotate and change the color of PDA. Although the blue-to-red transition mechanisms of PDA are not fully elucidated, it is widely accepted that the absorption characteristics of the main chain are very sensitive to the change of the strain. Theoretical calculation showed that only several degrees of rotation in the PDA main chain would significantly change the π-orbital overlap and affect the electronic state (Hao and Zhu, 2020). The color change of PDA is fast and highly sensitive. As a result, the current biological probes based on PDA are widely used in biological detection (Reppy and Pindzola, 2007; Yoon et al., 2009; Sun et al., 2010; Chen et al., 2012; Lee et al., 2016b; Qian and Stadler, 2019; Zhang et al., 2019a; Hao and Zhu, 2020). We here introduce the applications of PDA in colorimetric bio-detection according to the size and type of detection targets, including small biomolecules, biomacromolecules, microorganism, drug screening, etc.

### PDA Probes for Small Biomolecules

Small biomolecules, such as thiols, metal ions, amino acids, and fatty acids, are important components in organisms, and their concentration changes are often closely related to many diseases. For example, the lack of cysteine can lead to diseases such as tissue edema, lethargy, liver tissue damage and slow growth and development of infants (Yin et al., 2014; Zhang et al., 2017). L-arginine plays a key role in cell division, wound healing, immune function and hormone secretion, and low plasma and tissue arginine levels are important characters in septic patients (Zou et al., 2018). Calcium Ions are of great significance in various biological processes, including neurotransmitter release, synaptic plasticity, gene expression, mitochondrial metabolism, and programmed cell death (Zhao et al., 2019). Lysophosphatidic acid increase in the peripheral blood is observed in more than 90% of patients with early ovarian cancer, and thus lysophosphatidic acid in the peripheral blood is a promising biomarker for early detection of ovarian cancer (Wang et al., 2017). Since real-time, accurate, and highly sensitive detection of these small physiological and pathological molecules is of great significance to better understand the mechanism of the occurrence and development of physiological and pathological processes as well as the diagnosis and prevention of diseases, many PDA based probes have been developed in the detection of these small biomolecules (Cho E. et al., 2016; Cho Y. S. et al., 2016; Lee et al., 2016c, 2020; Park et al., 2016a; Kim et al., 2017; Li et al., 2017b, 2020; Zhang et al., 2018; Oh et al., 2019; Wang et al., 2019a).

**Figure 6:**
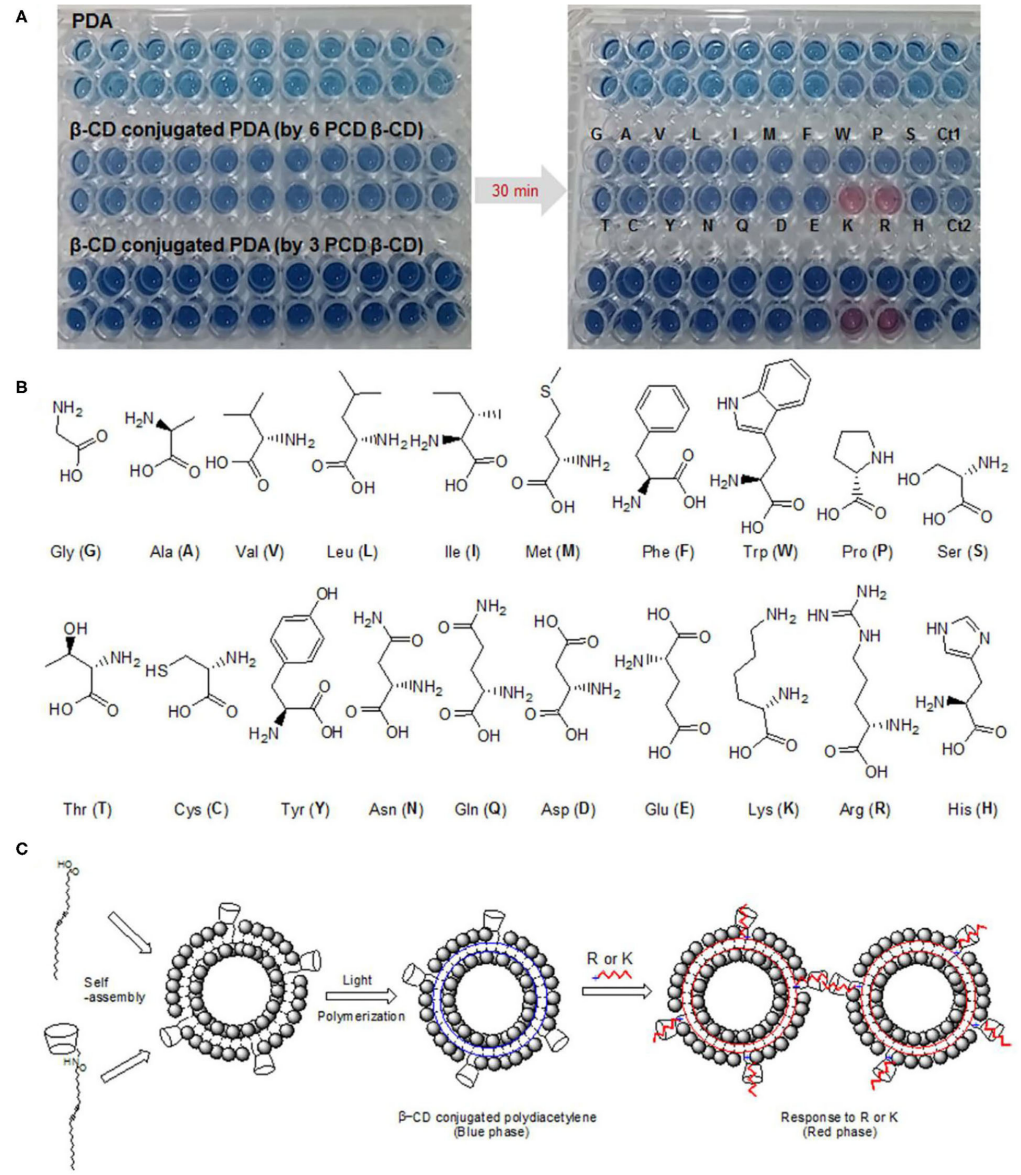
**(A)** Digital pictures of original PDA and β-CD-conjugated PDAs at room temperature after the addition of 20 amino acids. **(B)** The chemical structure of the 20 amino acids used. **(C)** Co-assembly of PCDA with β-CD to form β-CD-functionalized PDA and the schematic illustration of β-CD-conjugated PDA for arginine- and lysine-response. Reprinted with permission from Cho E. et al. (2016). Copyright 2016, Nature.

For example, Cho et al. developed the PDA vesicle functionalized with β-cyclodextrin to determine arginine and lysine (Figure 6) (Cho E. et al., 2016). The β-cyclodextrin head in PDA could effectively interact with the cationic arginine and lysine, making the color of PDA vesicles changed from blue to red. Oh et al. used PDA containing phosphate and carboxyl for detection of serum calcium ions (Oh et al., 2019). Calcium ions can form a strong coordination effect with the phosphate and carboxylate on the side chain of PDAs, which leads to changes in the arrangement of the side chains and changes in the color of PDAs. The PDA probes could effectively avoid the interference by Mg ions through the interaction of carboxyl and phosphate, thus demonstrating a good selectivity. Moreover, the probe had high SENSITIVITY and even detected calcium ions with a concentration as low as 0.97 μM in serum. Lee et al. developed a highly sensitive PDA probe toward biothiols (Lee et al., 2020). Diacetylene amphiphiles with pyridine groups undergone self-assembly mediated by mercury ions, and then polymerized under UV to yield a PDA probe for biothiols. Biothiols such as cysteine, homocysteine and glutathione could specifically bind to mercury ions on the PDA sensors, leading to the color change of PDA sensors. Our team also developed an imidazole-functionalized PDA probe for analysis of lysophosphatidic acid (Wang et al., 2017). The synergistic effect of hydrophobic interaction and charge interaction between the PDA probe and lysophosphatidic acid could selectively disturbed the electron cloud of the PDA backbone, triggering the color change of the PDA probes and realizing visualization detection of LPA in the peripheral blood with high sensitivity and specificity.

### PDA Probes for Biomacromolecules

Biomacromolecules, such as nucleic acids and proteins, play a variety of vital functions in organisms (Sun et al., 2018), such as carrying genetic information, catalysis, transport and storage of substances, mechanical support, exercise, immune protection, reception and transmission of information, regulation of metabolism and gene expression. PDA have also been applied for the detection of biomacromolecules in recent years (Zhang et al., 2016, 2019c; Wang et al., 2018a, 2019c; Jannah and Kim, 2019; Jung and Park, 2019; Lee et al., 2019b; Fang et al., 2020; Weston et al., 2020). According to the detection principles, PDA sensors for biomacromolecules can be divided into the following two categories.

The first one is designed according to the physicochemical properties and structural characteristics of biomacromolecules. For example, Zhang et al. developed an amine-terminated polydiacetylene vesicle to detect double stranded DNA (Zhang et al., 2016). They adopted wax screen printing to build paper-based reaction chambers, and then covered the test paper with amino functionalized PDA vesicles. The fast and sensitive colorimetric detection of double stranded DNA was realized by the positive and negative electric interactions between double stranded DNA and amino-functionalized PDA vesicles. With this method, the colorimetric analysis could be completed in 1 min with a low detection limit of 10 nM with the naked eye.

**Figure 7:**
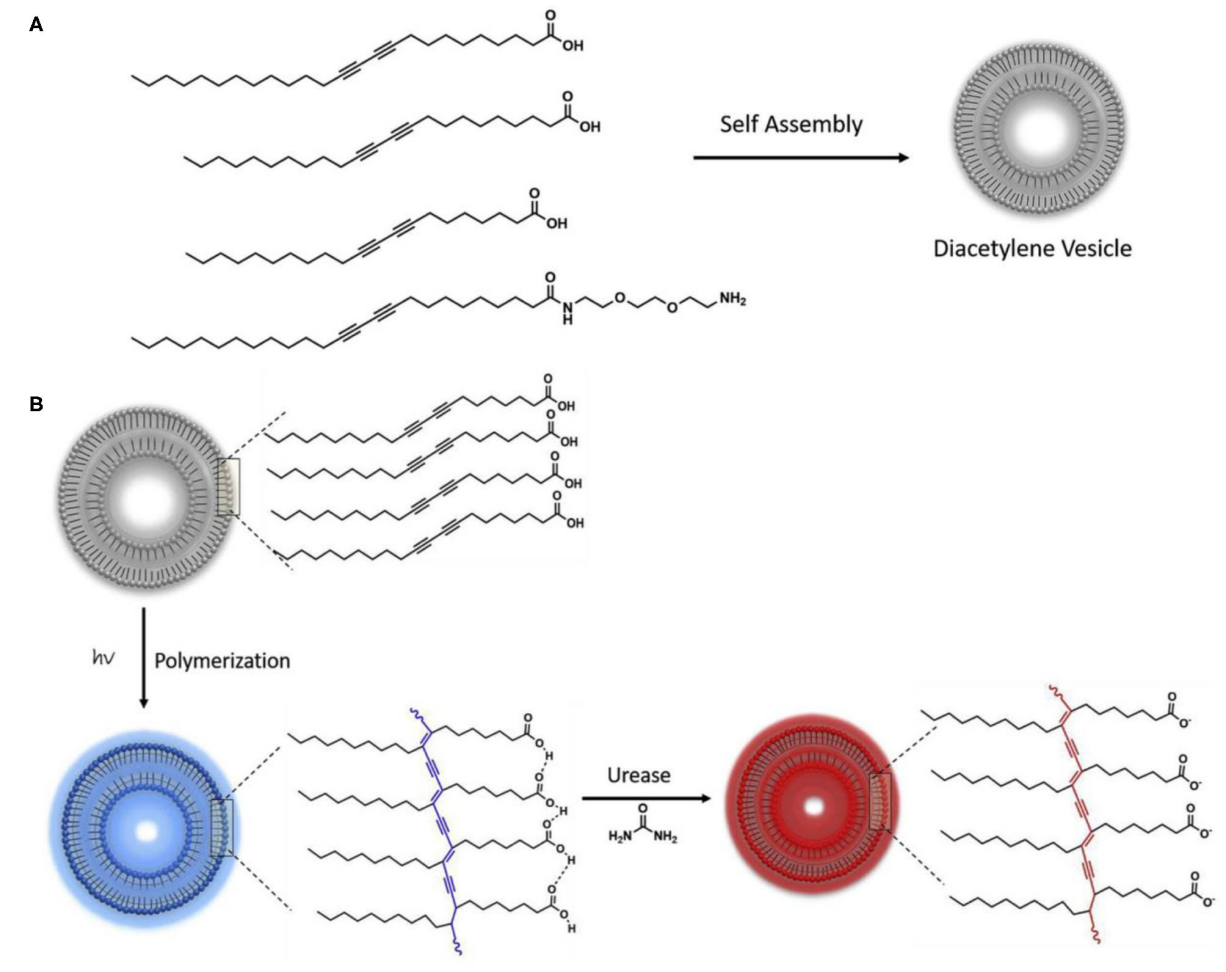
**(A)** Chemical structures of the diacetylene monomers including PCDA, TCDA, HCDA, and PCDA-EDEA. **(B)** Schematic illustration of PDA-based colorimetric sensing of urease. Reprinted with permission from Jannah and Kim (2019). Copyright 2019, Elsevier.

The other method involves utilizing the specific functions of biomacromolecules. For example, Yun Kyung Jun et al. developed a sensor based on glutathione-modified PDA liposomes to realize rapid and simple detection of glutathione S-transferase fusion protein (Jung and Park, 2019). Glutathione is known to be the substrate of glutathione S-transferase fusion protein. As a result, the interaction between glutathione S-transferase fusion protein and glutathione on the PDA liposomes could cause the color change of PDA backbones, thus realizing visual colorimetric detection of the target protein. Such strategy can also be used for monitoring the activity of enzyme through detecting the catalytic products. In another work, Zhang et al. developed a novel imidazole-functionalized PDA probe to analyze the activity of Photosholipase D by detecting phosphatidic acid, given the fact that photosholipase D can hydrolyze photoshadidylcholine to produce photoshadic acid (Zhang et al., 2019c). Negatively charged phosphatidic acids could interact with the imidazole groups on the side chain of PDAs to cause discoloration of PDAs. Jannah et al. also used similar strategy to detect urease activity by testing the concentration of ammonia, one of key hydrolysis products by urease (Figure 7) (Jannah and Kim, 2019). The catalytic product ammonia of urease leaded to the deprotonation of carboxyl group of the PDA side chains, which destroyed the hydrogen bond between the side chains of PDA and finally caused the change of the color of PDA probes.

### PDA Probes for Microorganisms

Charych et al. reported first example of using PDA for the detection of virus in 1993 (Charych et al., 1993). In recent years, PDA probes have been extended to detection of many microorganisms (Park et al., 2016c; Lebegue et al., 2018; Son et al., 2019). In general, PDA-based colorimetric probes toward microorganisms are prepared by modifying PDA with functional groups with specific affinity to microorganisms. For example, Seong UK Son and others developed a PDA probe for visual detection of pandemical influenza A virus (Son et al., 2019). They fixed PDAs on PVDF membranes, and then modified PDAs with antibodies specific to the pandemical influenza virus. The visual detection of pandemical influenza virus was realized through the specific binding between antibodies on the surface of PDA and the pandemical influenza A virus.

**Figure 8:**
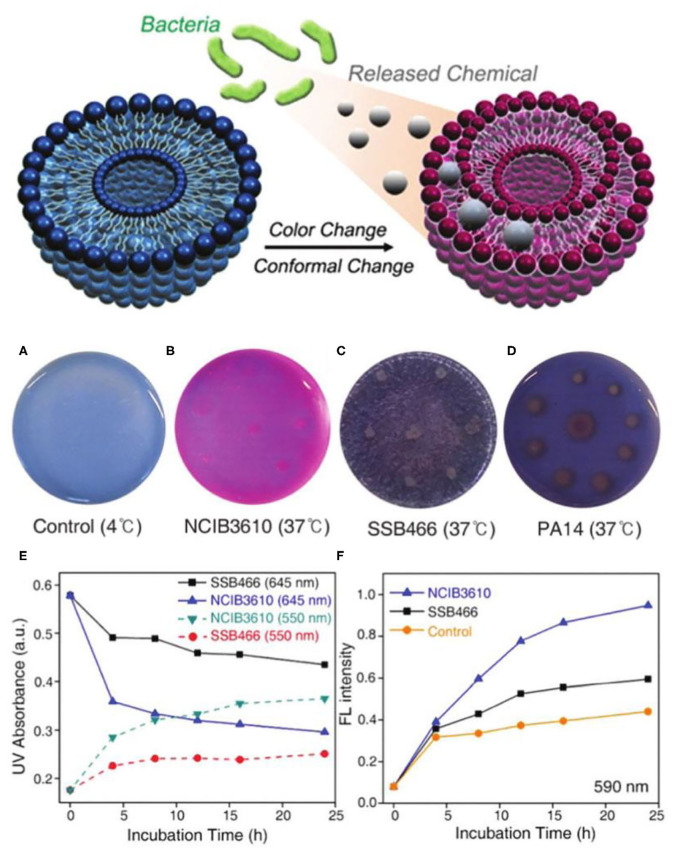
Bacteria-sensing mechanism of PDA based on the released chemical from bacteria. **(A–D)** Photometric change in PDA-LB-agar plate after incubation of various bacterial strains for 16 h. **(E)** UV/Vis and **(F)** fluorescence spectrum change of NCIB3610 and SSB466 incorporated PDA-LB-agarplate as function of incubation time with bacteria. Reprinted with permission from Park et al. (2016c). Copyright 2016, Royal Society of Chemistry.

Microorganisms can also be detected by biochemical substances released by pathogens. For example, Jimin Park and others presented a selective detection method by detecting the surfactant produced by Bacillus subtilis NCIB3610 (Figure 8) (Park et al., 2016c). Amino modified PDA liposome with the size of 30–80 nm were used to engineer probes of Bacillus subtilis NCIB3610. The probes were dispersed in LB medium containing Bacillus subtilis NCIB3610. The released surfactant to the LB medium from Bacillus subtilis NCIB3610 during growth and reproduction, led to the color change of the PDA probes. The negatively charged surfactant specifically interacted with the side chain of PDA by breaking the hydrogen bond between the amino groups. The PDA probes had a very good specificity, and only the microorganisms that could produce the surfactant can cause the blue-to-red color change of PDA, while no obvious color change was observed for other microorganisms.

### PDA Probes for Pharmaceutical Screening

Since PDA-derived colorimetric detection is fast, sensitive, and accurate, it has demonstrated great opportunities in high-throughput pharmaceutical screening and thus have attracted a wide range of interest from scientists and pharmaceutical companies in this field in recent years. It is very important for many drugs for mental or brain diseases to pass through the blood brain barriers, which are essentially a kind of liposome membranes. Based on this, Adhikary et al. prepared artificial blood brain barrier membranes containing PDA probes (Adhikary et al., 2020). By mixing diacetylene monomer 10,12-pentacosadiynoic acid and the polar brain lipid (contained phosphatidylinositol, phosphatidylethanolamine, as well as other lipoproteins, phospholipids, and neutral lipids), the artificial blood brain barrier membranes are obtained. After UV polymerization, the artificial membranes turn blue. When promethazine, hydroxyzine and other drugs penetrated this artificial membrane, the side chain arrangement of the PDA was destroyed and the color changes from blue to red. This method holds great promise for quick and high-throughput drug screening to treat brain diseases.

**Figure 9:**
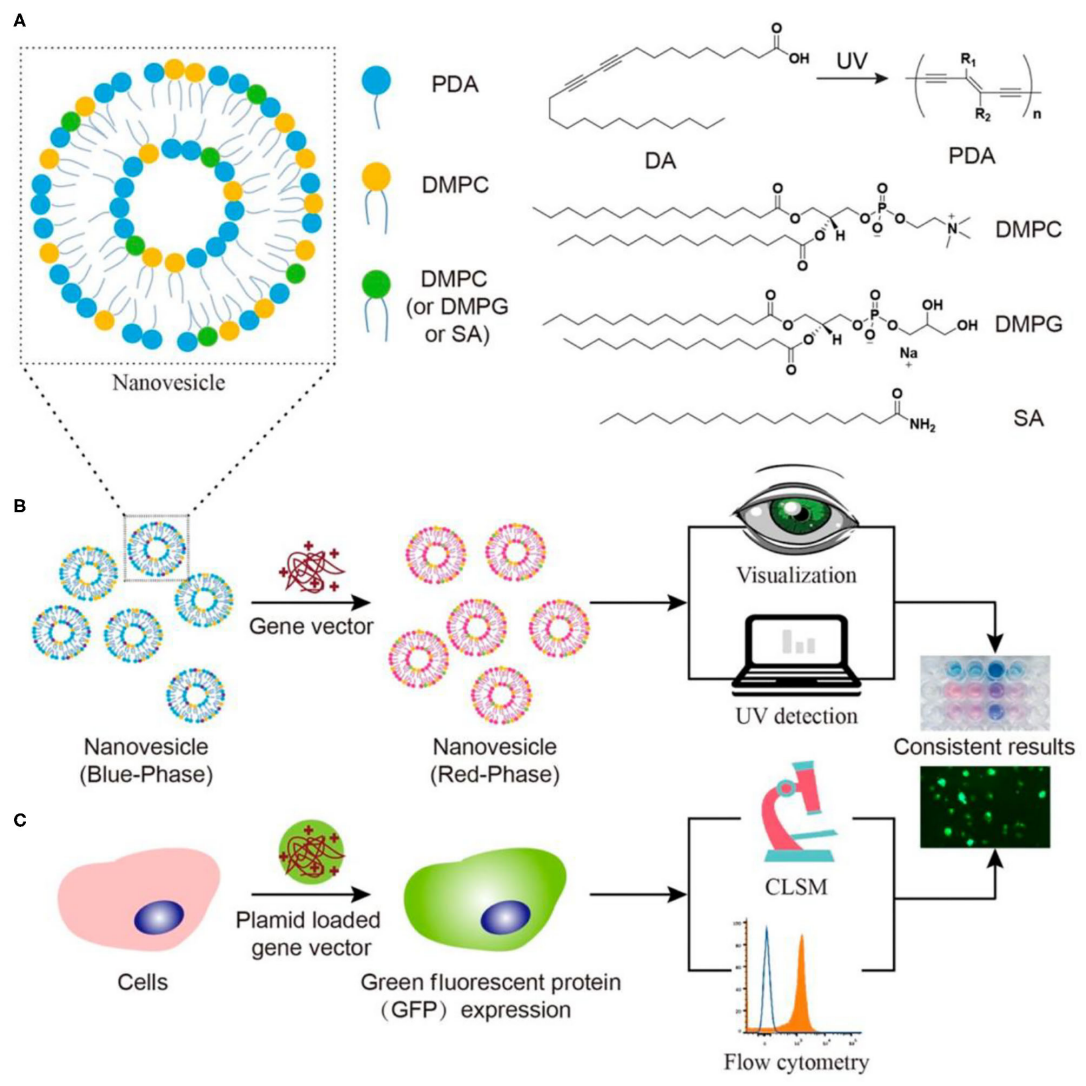
Schematic illustration of **(A)** the structure of nanovesicle composed of DA, PDA, DMPC, and/or DMPG/SA. **(B)** Fast visualization detection of the membrane affinity of gene vectors using PDA nanovesicles, compared to **(C)** a traditional cell transfection method. Reprinted with permission from Wang et al. (2019a). Copyright 2019, American Chemical Society.

In addition to drug screening, high-throughput screening of drug carriers is also important. However, the lack of powerful probes for monitoring the drug carrier efficacy significantly restricted the development of this field. To this end, Wang et al. developed a high-throughput, visual screening gene vector using PDA as a colorimetric probe. A series of artificial cell membranes were prepared by using PDA, 1,2-dimyristoyl-sn-glycero-3-phospho-1′-rac-glycerol, 1,2-dimyristoyl-sn-glycero-3-phosphocholine, and stearamide with different ratios or compositions. The color of the membrane would change if the affinity of the gene carrier to these artificial cell membranes was high (Figure 9) (Wang et al., 2019a). The process of penetrating artificial cell membranes would cause the side chain arrangement of PDAs to be destroyed and the color of PDAs to change. This rapid visualization method could not only predict the *in vitro* membrane affinity of gene carriers, but also provided a general strategy for high-throughput screening of various carrier materials with high cell affinity.

## PDA for Raman Detection and Raman Bioimaging

PDA are mainly composed of C-C-C, C=C, and C=C bonds, all of which have specific Raman scattering peaks. The intensity and position of Raman spectra of PDA derived from these specific bonds are closely related to the change of conjugated path length on the main chain of PDA. In the blue phase of PDA, the π-electron delocalization length of the main chain of PDA is large due to the restriction of the spatial arrangement of the side chain of PDA. The characteristic Raman peaks of C=C and C=C in PDA are located at 1,453 and 2,083 cm^−1^, respectively. When PDA changes from blue phase to red phase, the arrangement restriction of the side chain of PDA is released, resulting in a shorter π-electron delocalization length of the main chain of PDA and greater vibration amplitude of C=C and C=C. As a result, the C=C and C=C bond vibrations absorb more energy, and the Raman peaks of C=C and C=C move to the larger wave number. In the red phase of PDA, the Raman peaks of C=C and C=C shift to 1,515 and 2,123 cm^−1^, respectively (Wang et al., 2017).

Raman detection derived from PDA probes is very attractive in biological detection and imaging. Raman spectrum can provide rapid, simple, repeatable, non-destructive qualitative, and quantitative analysis. It does not need complicated sample pretreatment, and the samples can be measured directly through the optical fiber probe or through the transparent medium. Raman spectroscopy is particularly suitable for the detection or imaging of biological samples, because the Raman scattering of water is very weak, which can greatly eliminate the interference of the environment background. In addition, Raman detection has high selectivity, since molecules have their own Raman fingerprint peaks (Gan et al., 2019). However, such applications of PDAs are still in the infancy, presumably due to the weak Raman signal caused by the small Raman cross sections area of C=C and C=C in PDA (Jun et al., 2011; Kim H.-M. et al., 2015).

Recently, surface enhanced Raman scattering (SERS) and resonance Raman spectroscopy have been used to enhance the intensity of characteristic Raman signal of PDA. SERS technology can overcome the inherent signal limitation of traditional Raman spectrum. Its enhancement factor can be as high as 10^14^-10^15^ times, which is enough to detect the Raman signal of a single molecule. In 2018, Cui et al. assembled and polymerized PDA shell on the surface of silver nanoparticles (Cui et al., 2018b). They found that the characteristic Raman peaks of C-C-C, C=C, and C=C in PDA polymer were 83, 92, and 78 times higher than those in the thick PDA shell (about 20 ± 10 nm).

**Figure 10:**
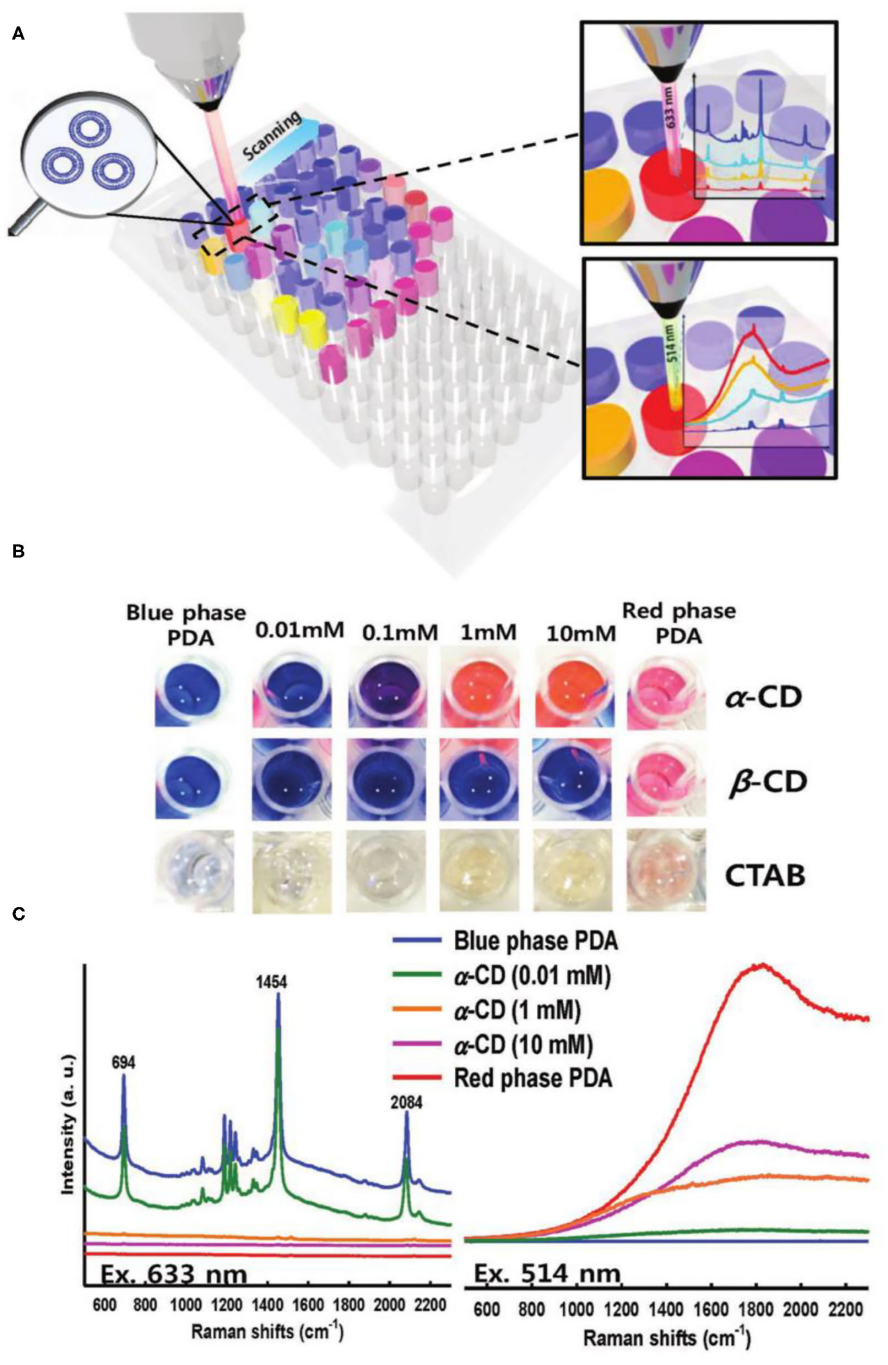
**(A)** A schematic representation of well-plate detection and **(B)** the corresponding optical images of PDA solutions upon exposure to α -, β -CD, and CTAB, respectively. **(C)** Raman spectra of PDA exposed to α –CD with laser excitation of 633 and 514 nm, respectively. Concentrations of the added CDs are indicated with lines of color. Reprinted with permission from Cui et al. (2018a). Copyright 2018, Wiley.

Compared with the SERS enhancement method, Resonance Raman Spectroscopy does not need to build the Surface Enhanced Raman Scattering base. Therefore, an drastic enhancement in the Raman intensity of PDA was also achieved by the Resonance Raman Spectroscopy technology to enhance the (Figure 10) (Cui et al., 2018a). When the wavelength of the incident laser matched well with the absorption wavelength of the conjugated polymer, Resonance Raman signals would be generated. The maximum enhancement ratio of the Raman signal could reach to 10^7^ times of the original. Specially, the maximum enhanced Resonance Raman can be obtained when the blue phase PDA is excited by 633 nm laser (the maximum absorption peak of the blue phase PDA). When PDA changed from blue phase to red phase, the excitation wavelength of PDA must be changed from 633 to 514 nm in order to obtain high Resonance Raman Spectroscopy enhancement effect. Raman detection of cyclodextrin was achieved by introducing ethylenediamine groups on the surface of PDAs. When cyclodextrin was present, the hydrogen bonding between the cyclodextrin and the side chain of PDAs caused the absorption of PDAs at 512 nm to increase greatly, and the Raman signal of PDAs could be greatly increased by 512 nm excitation. The PDA Raman probes based on the Resonance Raman Spectroscopy enhancement had very high sensitivity and could detect α-cyclodextrin at a low concentration of 0.01 mM.

It is worth noting that compared with other bioimaging methods, Raman imaging has the advantages of providing structure information of the detected biomolecules, narrow bandwidth, multi-color, multi-channel imaging analysis, among others. Proteins, nucleotides, lipids, sugars, and other biomolecules *in vivo* have strong background Raman signals in the range of 400–1,800 cm^−1^ and 2,800–3,100 cm^−1^. However, the Raman spectra of C-D, C=N and C=C bonds are located at 1,800–2,800 cm^−1^, outside Raman window of these biomolecules. Therefore, many molecules containing C-D, C=N and C=C such as ethynylbenzene, 5-ethynyl-2′-deoxyuridine, diphenyl phosphorazidate, diphenylbutadiyne, benzonitrile, and 5-bromopentaninitrile, have been developed for Raman imaging to minimize the background Raman signals *in vivo* (Yamakoshi et al., 2011; Song et al., 2014; Li et al., 2017c). However, these small-molecule Raman probes often have very weak Raman signals and thus not sensitive enough for *in vivo* Raman bioimaging. The chain of PDA contains C=C. More importantly, the super long π conjugation of PDA can increase the Raman resonance of C=C. Therefore, PDAs are considered promising candidates for creating effective Raman imaging probes.

**Figure 11:**
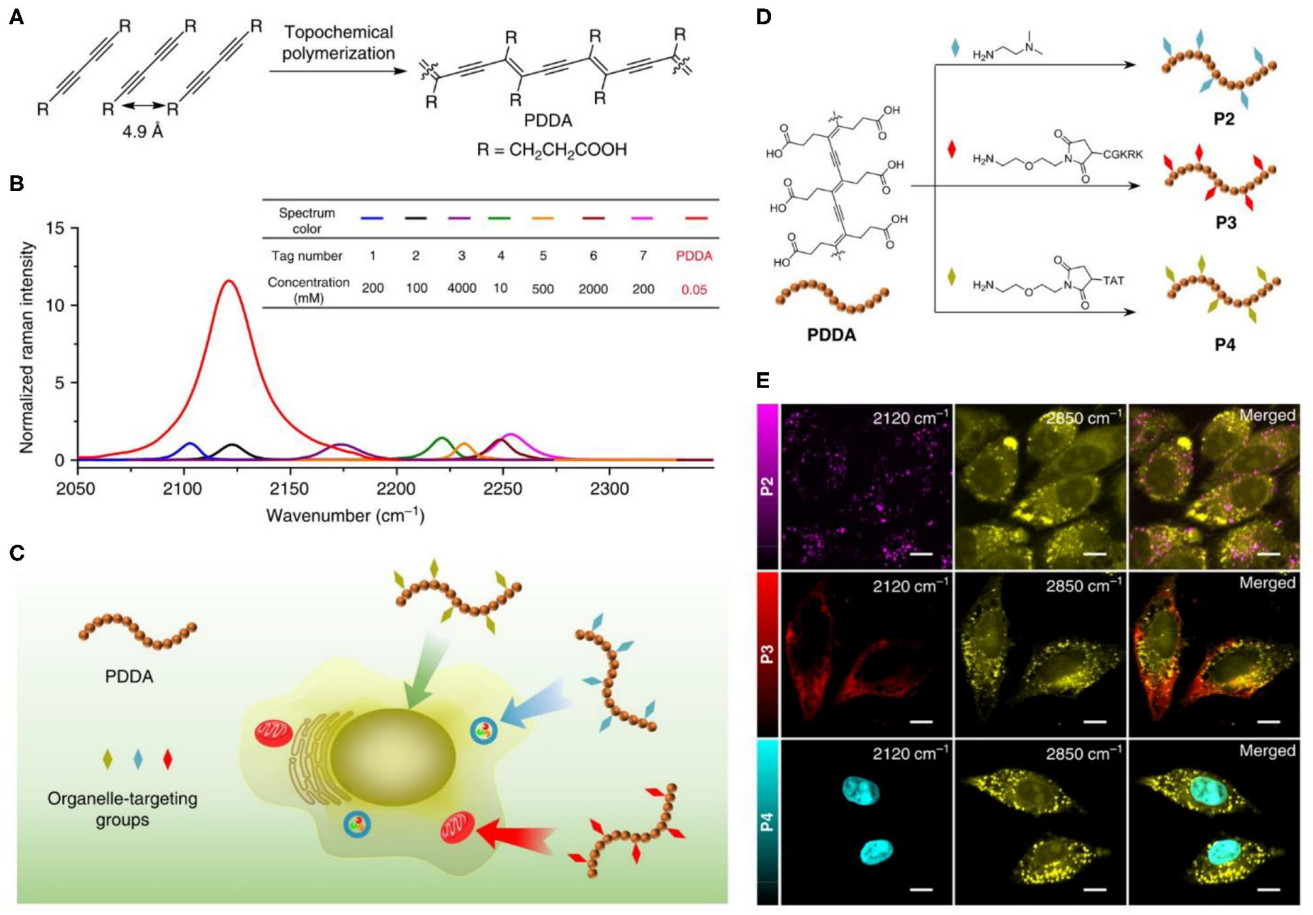
Polydiacetylene-based Raman probes for targeted live-cell Raman imaging. **(A)** Topochemical polymerization for the preparation of PDDA. **(B)** Overlaid Raman spectra of individual DMSO solution of PDDA and a series of representative Raman probes, including 1. ethynylbenzene; 2. 5-ethynyl-2′-deoxyuridine; 3. diphenyl phosphorazidate; 4. diphenylbutadiyne; 5. benzonitrile; 6. 5-bromopentanenitrile; 7.deca-4,6-diynedioic acid. **(C)** Schematic illustration of side chain modification of PDDA for subcellular organelle targeting Raman imaging. **(D)** Side chain modification of PDDA with different functional groups to derivatives P2, P3, and P4 that can be targeted to specific subcellular organelles. **(E)** Images of HeLa cells treated with 50 μM of P2, P3, and P4, respectively. Images shown from left to right are the alkyne (2,120 cm^−1^), lipids (2,850 cm^−1^), and merged images. Reprinted with permission from Tian et al. (2020). Copyright 2020, Nature.

Very recently, Tian et al. developed a PDA probe for Raman imaging of living cells (Figure 11) (Tian et al., 2020). the PDA Raman probe was prepared by self-assembly of deca-4,5-diynedioic acid and bis (pyridyl) oxalamide-functionalized PDA were self-assembled into an ordered structure via the host-guest interaction, followed by UV polymerization. They found that the Raman intensity of the C=C bond of PDA was different upon excitation at 488, 532, and 785 nm. Under 785 nm excitation, the average Raman intensity of each C=C was 100 times that of 5-ethylyl-2′-deoxyuridine, benefiting from the conjugate structure of the main chain of PDA. The C=C Raman signal of PDA could further be enhanced through the Resonance Raman mechanism when the excitation wavelength was closed to the absorption peak of PDA. The average each C=C Raman intensity of PDA through the Resonance Raman mechanism was even 10^4^ times higher than that of 5-ethylyl-2′-deoxyuridine under 488 and 532 nm laser excitation. Thanks to the special conjugate structure of PDA and the Resonance Raman mechanism, high quality Raman microscopic imaging of living cells could be realized by PDA. The ultra-strong alkyne Raman signal of PDA could be used as reporters for living cells Raman imaging. Through the specific functional modification of PDAs (P2-P4), the C=C Raman signal (2,120 cm^−1^) of PDAs can be used as a good Raman tag to image the lysosome (P2), mitochondrial (P3), and nucleus (P4) of living Hela Cells.

## PDA for Fluorescence Bio-Detection

Aside from the unique colorimetric property, the fluorescence of PDA has also attracted scientists' interest for a long time (Kobayashi et al., 1997; Lécuiller et al., 1999; Yamamoto et al., 2016; Roh et al., 2017; Cui et al., 2018b; Wang et al., 2018b, 2020; Kim and Lee, 2019; Kim et al., 2019). Similarly, fluorescence of PDA will change when PDA transformed from blue phase to red phase. Kobayashi et al. reported that the fluorescence lifetime of blue-phase PDA was only 0.92 and 1.08 ps at 290 and 10 K, respectively (Kobayashi et al., 1997). The ultrafast relaxation of the excited state of blue-phase PDA resulted in its ultra-weak fluorescence intensity. The quantum yield of the blue-phase PDA was lower than 1 × 10^−5^. In comparison, the fluorescence lifetime of red-phase PDA was 52 ps at 15 K, much longer than that of blue-phase PDA (Lécuiller et al., 1999). The long relaxation time of the excited state could greatly increase the fluorescence of red-phase PDA, with a quantum yield of about 0.3. However, the fluorescence intensity of red-phase PDA at room temperature was much weaker than that at 15K, and the quantum yield of red phase PDA was about 0.02. Of note, the quantum yield of red-phase PDA was still much higher than that of blue-phase PDA at room temperature. Therefore, PDA could be considered as a good “turn-on” fluorescent probe.

**Figure 12:**
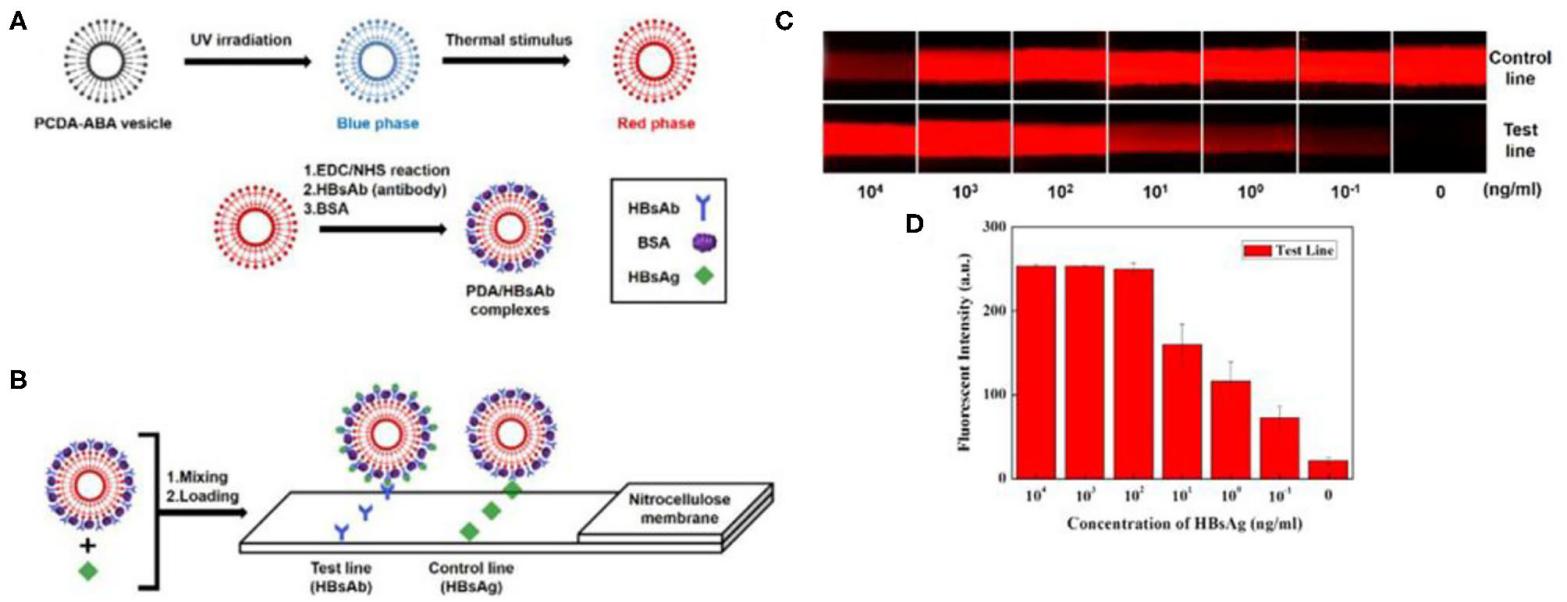
Schematic illustration of HBsAg detection on the NC membrane using red PDA/HBsAb complexes. **(A)** Schematic illustration of the structural design of PDA/HBsAb complexes for HBsAg detection. **(B)** Representative picture showing the detection of HBsAg on the NC membrane using red HBsAg- PDA/HBsAb complexes. Detection of HBsAg using PDA/HBsAb complexes. **(C)** Fluorescent image of the NC membrane (exposure time:1/3.5 s) and **(D)** fluorescent quantitative analysis of the test line. Reprinted with permission from Roh et al. (2017). Copyright 2017, Wiley.

The PDA-based fluorescent probes had many merits, such as high sensitivity, good selectivity, wide linear range, and little influence by external conditions. Therefore, PDA fluorescent probes have been widely used in biomolecular detection and cell imaging analysis in recent years (Wu et al., 2014; Zhang et al., 2014, 2018; Jiang et al., 2017). For example, Roh et al. developed a PDA-based fluorescent probe for hepatis B surface antigen (Figure 12) (Roh et al., 2017). Moreover, a convenient and economical fluorescent probe was prepared by loading the Hepatitis B surface antibody-modified PDA onto nitrocellulose test paper. In the presence of hepatitis B surface antigen, PDA would undergo blue-to-red phase transformation and emitted red fluorescence. In this way, the detection of hepatitis B surface antigen could be realized by both fluorescence and colorimetric detection with high sensitivity. However, the lowest detection limit of hepatis B surface antigen by the fluorescence method was 0.1 ng/ml, 10 times lower than that of colorimetry (1 ng/ml) under the same conditions, suggesting the higher sensitivity of PDA fluorescent probes.

To some extent, the low quantum yield of PDA fluorescence at room temperature limits applications of PDA fluorescence probes for biological detection and bioimaging. To address this limitation, Cui et al. developed an effective way to enhance the fluorescence of PDA by combining metal nanoparticles with surface plasmon enhancement effect (Cui et al., 2018b). They prepared Ag@PDA nanoparticles with a core-shell structure. The interaction between silver nanoparticles and PDA not only made the maximum fluorescence emission peak of PDA red shifted by 10 nm, but also made the fluorescence intensity of Ag@PDA nanoparticles enhanced by 7 times compared to pure PDA.

**Figure 13:**
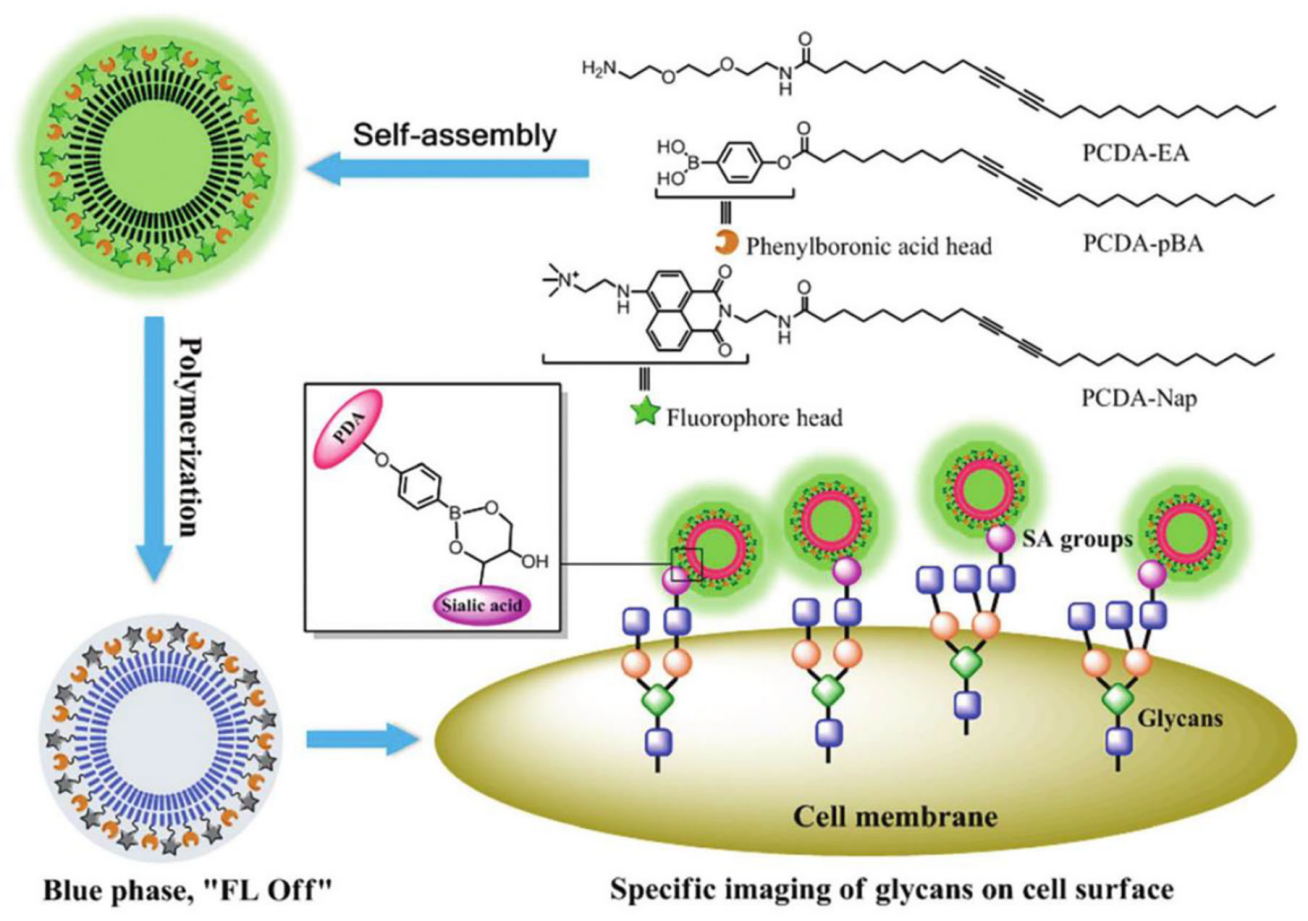
Self-assembly of PCDA-EA, PCDA-pBA and PCDA-Nap to form the composite PDA liposomes and the schematic illustration of the as-prepared PDA liposomes for specific cell-glycan imaging. Reprinted with permission from Wang et al. (2018b). Copyright 2018, Royal Society of Chemistry.

Apart from metal, the introduction of fluorescence energy resonance transfer mechanism into the detection can improve the fluorescence quantum yield of PDA. For example, Wang et al. prepared a fluorescent sensor containing PDA liposome composite for sialic acid detection by assembling and polymerizing three kinds of amphiphilic diacetylenes with the head group of phenylboric acid, 1,8-naphthalimide derivatives and ethylenediamine (Figure 13) (Wang et al., 2018b). In this PDA fluorescence sensor, the phenylboric acid head group could specifically bind with sialic acid and 1,8-naphthalimide derivative (the quantum yields >0.4) were fluorescence indicators. In the blue phase of PDA liposomes, the energy transfer between the fluorescence group of 1,8-naphthalimide derivative and the conjugated skeleton of PDA could quench the fluorescence of 1,8-naphthalimide derivative. In the presence of sialic acid, nevertheless, the blue-phase PDA would transform into the red-phase PDA, resulting in the fluorescence recovery of 1,8-naphthalimide derivative. The PDA fluorescence sensor had very high sensitivity, with the minimum detection limit of 14 μM. Considering that sialic acid locates at the end of cell membrane glycan an play an important role in many biological and pathological processes, these authors further extended the use of PDA fluorescent probes for analysis of SA on the surface of living cells (Wang et al., 2020).

## Conclusion and Prospect

In this review, we summarized the progress of PDA in the field of biological detection and bioimaging. Since the preparation of PDA have a great influence on the performance of the PDA biosensors, we first introduced the latest progress of PDA preparation technology in recent years, including PDA nanomaterials, 3D-PDA, porous PDA, chiral PDA, reversible response PDA and so on. Next, the applications of PDA for biological detection and bioimaging were discussed according to their optical properties. The characteristics and the latest research progress of three types of PDA probes including colorimetry, fluorescence, and Roman were overviewed. In the colorimetric PDA probes, we discussed the source and influencing factors on PDA's absorption and then provided recent progress of colorimetric PDA probes in the biological detection according to the size of targets. In terms of PDA Raman probe and fluorescence probe, we analyzed the origin and influencing factors of Raman scattering and fluorescence, as well as compared the advantages and disadvantages of these two types of probes in biological detection and bioimaging.

PDA probes have great potential *in vitro* convenient detection such as Point-of-care testing (POCT). PDA can produce distinguished color change from blue to red, which can be easily recognized by human eyes. Therefore, PDA has a great prospect in many fields, such as convenient biological detection, test paper detection, high-throughput detection, among others. However, the signal stability, selectivity and preparation methods of PDA still need further improvement. At present, most PDA bioprobes are in the stage of laboratory investigation. By the cooperation of multi-disciplinary scientists, we believe that the clinical applications of PDA probes in biomedicine will be realized in the future.

Although PDA has great advantages in biomedicine, most of the current applications are carried out *in vitro* or at the cell level, and its application in real *in vivo* applications is still limited. PDA can not only be used as a drug carrier, but also has special optical properties such as absorption, fluorescence, and Raman. Therefore, PDA should be very promising in terms of intelligent biomedical materials. We believe that in the near future, PDA smart materials that integrate controlled drug release, specific imaging and detection can truly be used *in vivo*.

## Author Contributions

KA and QH designed the framework of the reviews. All authors wrote the manuscript.

## Conflict of Interest

The authors declare that the research was conducted in the absence of any commercial or financial relationships that could be construed as a potential conflict of interest.
